# Psuedomyxoma peritonei secondary to adenocarcinoma of the cecum

**DOI:** 10.4103/1319-1683.71993

**Published:** 2010

**Authors:** Hussah Al-Buainain, Yasser Al-Jehani, Khaled Moghazy, Abdulaziz Al-Quorain

**Affiliations:** *Department of Surgery, University of Dammam, Dammam, Kingdom of Saudi Arabia*; 1 *Department of Radiology, University of Dammam, Dammam, Kingdom of Saudi Arabia*; 2 *Department of Internal Medicine, University of Dammam, Dammam, Kingdom of Saudi Arabia*

**Keywords:** Pseuomyxoma peritonei, mucinous adenocarcinoma

## Abstract

Pseudomyxoma peritonei is a rare progressive disease. Patients commonly present with a picture of acute appendicitis or with increasing abdominal girth. We present a case of a 71 year old man who presented with right iliac fossa pain, fever and vomiting. His abdominal examination revealed right iliac fossa mass which was confirmed radiologically. Diagnostic laparoscopy showed jelly like material along with a right iliac fossa mass. The aspirate was negative for malignancy initially. Due to persistance and progression of his disease he underwent right hemicolectomy. Histopathological diagnosis showed moderately differentiated adenocarcinoma of the cecum Duke’s C2.

## INTRODUCTION

Pseudomyxoma peritonei (PMP) is a poorly understood condition affecting both sexes equally, with an increasing incidence with age.[1] It is characterized by progressive intraperitoneal mucinous tumor and ascites. Recent immunohistochemical findings have added strong support to the theory that most tumors associated with PMP originate from the appendix.

The quantity and distribution of the tumor can be determined by redistribution phenomenon.[2] Preoperative diagnosis has often been as appendicitis or ovarian tumor.[3] Laparoscopic approach plays an important role in the diagnosis and the management of such a condition.[4] The rarity of this condition and its heterogeneity has led to the lack of unified protocols regarding its treatment. The preferable treatment is a combination of cytoreductive surgery and perioperative chemotherapy. The majority of patients develop recurrence which requires repeated surgical intervention.[5]

## CASE REPORT

This is a case of a 71 year man who is known to have coronary artery disease, hypertension and atrial fibrillation and is on medications. He presented to the emergency department of King Fahd Hospital of the University with localized right iliac fossa pain which was associated with fever, nausea and vomiting. There was no change in the bowel habit and there was no mucus or blood in his stool, he also didn’t have any urinary symptoms. He denied any history of weight loss. His past history was significant for cardiac catheterization, right inguinal hernia repair, upper GI endoscopy and frequent admissions for uncontrolled atrial fibrillation. Systemic review was unremarkable. The physical examination revealed an ill looking patient with slight tachycardia and low grade fever. His respiratory and cardiovascular systems were within normal limits.

The abdomen revealed a right iliac fossa mass measuring 8 × 6 cm firm, fixed, tender, not pulsatile and no skin changes over it. There was no organomegaly or palpable lymphadenopathy.

Laboratory investigations showed leucocytosis of 13.6 with neutrophilia.

The carcinoembryonic antigen (CEA) 54.1 (0-3ng/ml) was elevated, other laboratory investigations including Alpha-fetoprotein (AFP) were within normal limits.

**Figure 1:**
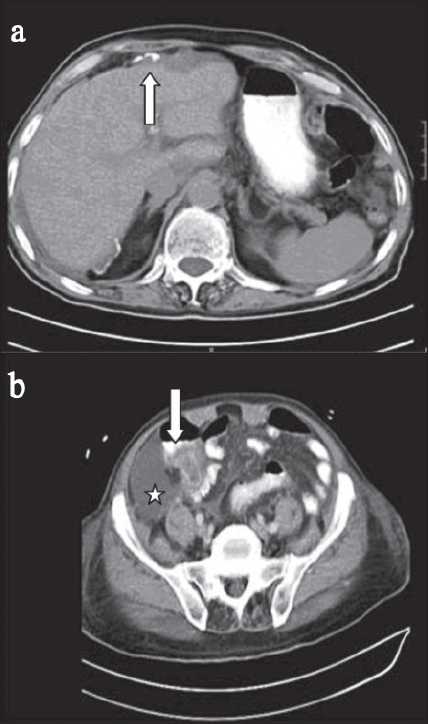
Axial CT scan of the abdomen and pelvis with oral and IV contrast (a) Intraperitoneal fluid collection seen at the right subdiaphragmatic space with calcifications (arrows). (b) Heterogeneous mass at the cecum and appendix (open arrow) with free fluid collection (*)

**Figure 2:**
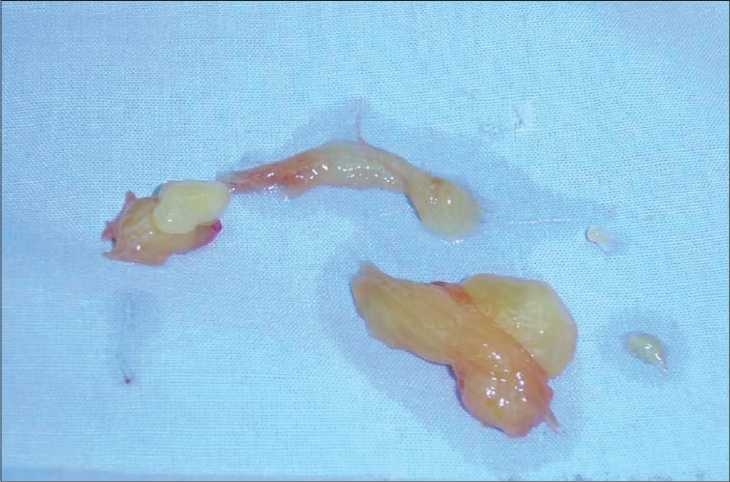
Yellowish brown jelly like material in abdominal cavity

Abdominal sonography showed minimal fluid collection in the subhepatic area and a complex mass in the right iliac fossa. CT scan of the abdomen and pelvis with double contrast showed a heterogeneous soft tissue mass on the medial aspect of the cecum and ascending colon with central necrosis [Figure 1]. Diagnostic laparoscopy revealed free yellowish brown jelly like material in the abdominal cavity which was aspirated and sent for analysis [Figure 2].

**Figure 3:**
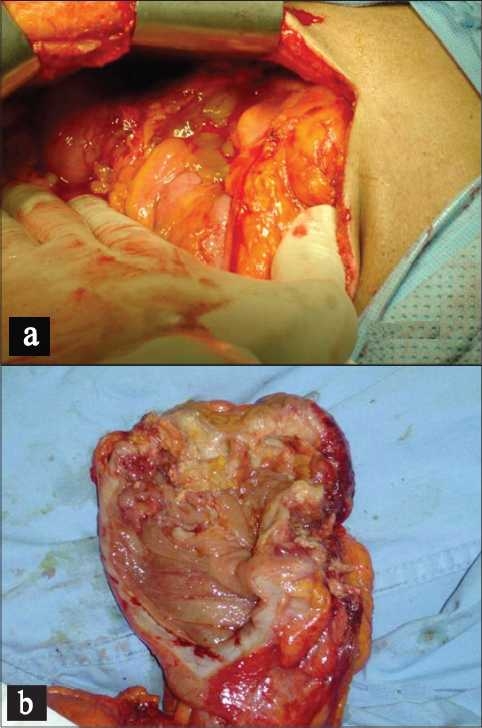
(a) Jelly like material in the abdominal cavity. (b) Right iliac fossa mass involving terminal ileum, cecum and appendix

A fixed mass was seen in the area of the cecum, the appendix was not visualized. The patient had an uneventful post diagnostic laparoscopy and he was discharged upon his request. The histopathology evaluation confirmed the presence of areas of mucus, focal hemorrhage and calcifications with fibrofatty tissue but no clear evidence of malignancy. The patient presented 3 months later to the emergency department with right iliac fossa pain similar to the previous attack but it was more severe. He was looking ill, febrile and tachycardic. The laboratory test showed leucocytosis with neutrophilia. Repeated CT scan revealed an increase in the size of the mass. The patient agreed on surgical intervention. Intraoperatively the mass was found involving the terminal ileum and attached to the posterior abdominal wall. Complete excision of the mass was achieved followed by right hemicolectomy [Figure 3]. The patient had an uneventful postoperative course and was discharged in a satisfactory condition with follow up with the oncology division. The histopathology revealed a moderately differentiated mucinous secreting adencarcinoma with 4/7 lymph nodes and free margins, Duke’s stage C2. Unfortunately, the patient developed massive stroke and passed away shortly after his discharge.

## DISCUSSION

PMP is a rare progressive disease, characterized by the production of copious amounts of mucinous fluid that gradually fills the peritoneal cavity, resulting in the so called ‘jelly belly’ abdomen.[2] The first case was described by Rokitansky in 1842,[6] but the term PMP was described in 1884 in association with a mucinous carcinoma of the ovary.[5] Later on, In 1901 Frankel[7] described PMP in association with a cyst of the appendix.

Due its rarity and the small number of published cases, the pathological origin and the ideal management of this condition is a subject of debate.

This condition is thought to originate from appendiceal adenoma which occludes the lumen of the appendix followed by gradual distention of the distal part with mucus and epithelial cells till its ruptures. The location and quantity of mucinous tumor within the abdomen and pelvis is characterized by a redistribution phenomenon that accumulates along the absorptive area of peritoneal fluid e.g greater omentum and diaphragm. In contrast the visceral peritoneum of the small bowel are seeded sparsely.[2,8]

The massive accumulation of tumor cells within the greater omentum contributes to the increase of the abdominal girth which is one of the common presentations.

In some instances the appendiceal distention and rupture, result in bacterial contamination and the patients present with symptoms of acute appendicitis.

It’s worth mentioning that the literature contains sporadic cases of PMP in association with tumors of GI, lung, breast, pancreas, gallbladder, bile duct, fallopian tubes, ovaries, and urinary bladder.[5]

Recent immunohistochemical findings have added strong support to the theory that most tumors associated with PMP originate from the appendix.

Due to the different pathological terms PMP is considered as a clinico-pathological entity characterized by mucinous ascites and non-invasive mucinous implants with a characteristic distribution. It contains histologically benign mucinous epithelium derived from an appendiceal mucinous adenoma which were labeled as ‘disseminated peritoneal adenomucinosis’ (DPAM), whereas similar cases with malignant histological features, were classified as ‘peritoneal mucinous carcinomatosis’ (PMCA). These peritoneal mucinous carcinomatosis with intermediate or discordant features were labeled as (PMCA-I/D).[5]

PMP is a subclinical event which is usually diagnosed at laparoscopy or laparatomy for appendicitis or ovarian tumor. The most likely explanation is that the appendix may be repeatedly decompressed by perforation and resealing. The most commonly recorded symptoms are abdominal pain resembling acute appendicitis in 27%, distension or mass in 23%.[3] Nausea, vomiting and fatigability have been described.[9] In rare cases the mucinous collection can lead to signs of raised intraabdominal pressure, such as uterine prolapse or hernia.[10]

Imaging studies before surgery is highly desirable, since a preoperative diagnosis allows the operation to be planned well and performed accordingly. Plain films are generally considered unhelpful for the diagnosis but the CT scan is widely employed to establish the diagnosis and extent of PMP. The mucinous material appears heterogeneous, scalloping of the liver, spleen and mesentery with central displacement of bowel loops and occasional calcifications. Sugarbaker *et al*.[2] used CT scan preoperatively to distinguish peritoneal adenomucinosis from peritoneal mucinous carcinomatosis depending on the tumor size and focal narrowing of the small bowel loops with segmental obstruction.

The peritoneal aspirates typically contain predominantly mucus and few cells and the mucus stains positively for periodic acid–Schiff, astra blue, mucicarmine and alcian blue. It is impossible to assess the sensitivity and specificity of aspiration cytology in the diagnosis of PMP, hence, its value in clinical practice remains uncertain. Elevated tumor markers such as CEA level, CA19-9 and CA125 indicate advanced disease, and may rise in association with recurrent disease.[3]

In the past the treatment consisted of repeated debulking surgery which is not curative but aims to resect gross disease and limits mucus production and accumulation. Sugarbaker *et al*.[2] advocated ultra-radical surgery consisting of peritonectomy, great omentectomy, splenectomy, stripping of right and left hemi diaphragm, cholecystectomy, lesser omentectomy, antrectomy and pelvic peritonectomy. In addition the excision of rectosigmoid, right hemicolectomy, heated intraperitoneal chemotherapy for 90 minutes and systemic chemotherapy were also recommended.[11] These procedures are associated with a lot of complications as observed by Cough *et al*.[9] There are some aids to operative cytoreduction, such as dextrose solution and mucolytic agents,[12] in addition to the use of photosensitization and laser, but they don’t seem to be very beneficial.

In the Mayo clinic series, survival was significant higher in patients who received intraperitoneal chemotherapy than those given systemic chemotherapy.[9] Recently, laparoscopy allows abdominal exploration, irrigation and aspiration of the ascetic fluid. In addition to this hemicolectomy can be performed along with catheter placement for chemotherapy.[4]

Recurrence of the disease may take years to develop or become symptomatic. Surgical reintervention is usually difficult because adhesions and fibrosis greatly increase the risk of unintentional enterotomies with subsequent leaks and fistulae formation. it is however, widely accepted that recurrences should be investigated vigorously and treated with further surgical debulking, with or without adjuvant chemotherapy, in the expectation that many patients will enjoy substantial additional survival and free of symptoms.

In the Mayo Clinic series,[9] the median follow-up period was 12 years (9–25·6), patient survival was considerably better than in other observation with 76% of patients developed recurrence and 50% of recurrences occurred within 2·5 years. Sugarbaker *et al*.[2] reported that approximately 33% of their patients required reoperation for recurrent adenomucinosis.

Recurrent disease occurs on the bowel surfaces and it is associated with intense fibrosis and adhesions leading to intestinal obstruction and obstructive jaundice, which are often the cause of death in PMP.[3] Metastases to lymph nodes and to the parenchyma of solid organs are unusual and represent relatively high-grade mucinous carcinoma.

In the Mayo Clinic series,[9] median survival of 5·9 years, and 5- and 10-year survival rates of 53% and 32% respectively were reported in 56 patients. As reported by Ronnett *et al*.[13] the survival is considerably worse in patients with more malignant, clinical and histological features.

## CONCLUSION

PMP is a rare and heterogeneous disease. Its pathological origin is uncertain and ideal treatment is a subject of debate. Mucinous adenocarcinoma of the cecum can mimic this condition and add to diagnostic difficulties. The role of diagnostic laparoscopy in such doubtful cases is valuable. The management requires an accurate preoperative assessment and diagnosis. Recent application of immunocytochemistry and genetic analysis using polymerase chain reaction have made major contributions to the debate.
